# Bioremediation in marine ecosystems: a computational study combining ecological modeling and flux balance analysis

**DOI:** 10.3389/fgene.2014.00319

**Published:** 2014-09-12

**Authors:** Marianna Taffi, Nicola Paoletti, Claudio Angione, Sandra Pucciarelli, Mauro Marini, Pietro Liò

**Affiliations:** ^1^Department of Biosciences and Biotechnology, University of CamerinoCamerino, Italy; ^2^Department of Computer Science, University of OxfordOxford, UK; ^3^Computer Laboratory, University of CambridgeCambridge, UK; ^4^National Research Council (CNR), Institute of Marine Sciences (ISMAR)Ancona, Italy

**Keywords:** ecological network analysis, flux balance analysis, bioremediation, PCBs, *Pseudomonas putida*, Adriatic sea

## Abstract

The pressure to search effective bioremediation methodologies for contaminated ecosystems has led to the large-scale identification of microbial species and metabolic degradation pathways. However, minor attention has been paid to the study of bioremediation in marine food webs and to the definition of integrated strategies for reducing bioaccumulation in species. We propose a novel computational framework for analysing the multiscale effects of bioremediation at the ecosystem level, based on coupling food web bioaccumulation models and metabolic models of degrading bacteria. The combination of techniques from synthetic biology and ecological network analysis allows the specification of arbitrary scenarios of contaminant removal and the evaluation of strategies based on natural or synthetic microbial strains. In this study, we derive a bioaccumulation model of polychlorinated biphenyls (PCBs) in the Adriatic food web, and we extend a metabolic reconstruction of *Pseudomonas putida* KT2440 (iJN746) with the aerobic pathway of PCBs degradation. We assess the effectiveness of different bioremediation scenarios in reducing PCBs concentration in species and we study indices of species centrality to measure their importance in the contaminant diffusion via feeding links. The analysis of the Adriatic sea case study suggests that our framework could represent a practical tool in the design of effective remediation strategies, providing at the same time insights into the ecological role of microbial communities within food webs.

## 1. Introduction

Aquatic ecosystems are subject to a mixture of synthetic organic chemicals, leading to adverse effects on organisms at different levels of biological organization and at all trophic levels of the food web. Over the last decades, many removal strategies have been proposed in order to reduce the bioavailability of persistent organic pollutants (POPs) and to limit the consequent bioaccumulation phenomena on species. *Polychlorinated biphenyls* (PCBs) are a class of POPs consisting of 209 different congeners, obtained from the catalytic chlorination process of biphenyl, and characterized by high environmental persistence and resistance to natural ways of breakdown. PCBs are practically insoluble in water and, due to their lipophilic nature, they easily dissolve in fats and lipids causing bioaccumulation, i.e., the phenomenon by which the internal contaminant concentration in an organism is higher than in the external medium. Indeed, PCBs have been detected both in aquatic biota and in all the abiotic phases of marine environments (sediments, water and dissolved organic carbon). Generally, heavier chlorinated PCBs congeners tend to accumulate in oxygen-depleted zones of sediments. Moreover, they bioconcentrate in species by following biomass flows in predator-prey relationships. PCBs bioccumulation phenomena in aquatic organisms occur over time as the result of multiple contamination pathways, including processes of uptake (e.g., dietary and dermal absorption) and elimination (e.g., egestion and respiration).

However, not all the living organisms in a polluted environment are prone to bioaccumulation. The sizeable variety of marine microbial life is metabolically involved in many transformation processes like biogeochemical cycles of elements, water quality conservation and biodegradation of many organic pollutants. Microbial communities are also an active compartment at the lower trophic levels of marine food webs. They interact with the grazing activities of planktonic groups and play a crucial role in the mineralization of organic matter through the complex trophic pathway known as the *microbial loop* (Fenchel, 2008). The bioremediation of PCBs is biologically incomplete, since it takes place via two distinct microbially mediated processes: anaerobic bacteria by reductive dechlorination remove chlorine atoms in higher chlorinated congeners, which are then oxidatively reduced by aerobic bacteria via cometabolic reactions (Brown et al., 1987; Bedard and Quensen, 1995). Even if PCBs are difficult to fully degrade, the patterns of PCBs mixtures can potentially lead to the development of novel catabolic pathways, thus increasing the genetic microbial variability in the aquatic ecosystem (Pieper and Reineke, 2000; Lovley, 2003).

Computational models and predictive tools have found wide applicability and usefulness both in ecotoxicological studies and in the reconstruction of genome-scale metabolic network of pollutant degrading bacteria. However, to the best of our knowledge, these techniques have never been considered for investigating, in a combined way, the multiscale effects of microbial bioremediation at the ecological level. In this work, we develop a computational framework that integrates bioaccumulation information at ecosystem level with genome-scale metabolic models of degrading bacteria. We apply it to the case study of the PCBs bioremediation in the Adriatic food web.

Specifically, we estimate the PCBs bioaccumulation model by using Linear Inverse Modeling, and we employ Flux Balance Analysis to extend the metabolic reconstruction of the toluene degrading bacteria *Pseudomonas putida* KT2440 (iJN746), presented in Nogales et al. (2008), with the aerobic pathway of PCBs degradation. We also provide a general method to obtain integrated ecological-metabolic models, relying on a reaction-based encoding of the food web and on the definition of different bioremediation scenarios. We analyse the effects of varying oxygen levels on the microbial growth and on the PCBs uptake of the extended metabolic network of *P. putida* by means of bilevel optimization to evaluate the efficiency of biomass production when PCBs uptake is favored and when interactions with the toluene degradation pathway are considered. Finally, we apply ecological network analysis tools to study structural properties of the bioaccumulation networks obtained at increasing degrees of bioremediation efficiency. By testing different bioremediation interventions, our computational experiments provide insights into the potential reduction of bioconcentration in the food web, into the role of species in the diffusion of PCBs, and ultimately, into the overall status of ecosystem sustainability.

## 2. Methods

### 2.1. Estimation of PCBs bioaccumulation in the adriatic sea

In our framework we focus on the case of PCBs bioaccumulation in the Adriatic sea, a semi-enclosed basin characterized by high biodiversity (Coll et al., 2010; Danovaro et al., 2010) and by the presence of multiple contamination sources and anthropogenic perturbations. In the last decades different species of ecological and commercial interest have been surveyed in this region and several toxicological studies report the occurrence of PCBs bioaccumulation in the Adriatic sea (Corsolini et al., 2000; Bayarri et al., 2001; Marcotrigiano and Storelli, 2003; Perugini et al., 2004; Storelli et al., 2007; Sagratini et al., 2008). We consider the PCBs bioaccumulation model presented in (Taffi et al., 2014) where a review of bioaccumulation studies in the North, Central and South Adriatic sea (period 1994–2002) is conducted in order to estimate bioconcentrations and PCBs flows among species. The model consists of 39 functional groups and is defined on top of a trophic reconstruction obtained from data collected in Coll et al. (2007), one of the most complete quantitative studies of the Northern and Central Adriatic food web.

We assume that organic chemicals follow the same paths as biomasses, moving via feeding link through the trophic structure of the food web, which is a common approach in the field of ecotoxicological modeling (Hendriks et al., 2001; Christensen and Walters, 2004; Laender et al., 2009). Flow rates quantify the intensity at which the contaminant is transferred from the source to the target (i.e., from prey to predator), and are estimated at mass-balance conditions from bioconcentration and biomass values of the involved groups. We also include external unbalanced compartments, implementing potentially unlimited exogenous imports and exports. Network estimation is achieved through *Linear Inverse Modeling* (LIM) (van Oevelen et al., 2010), used to compute flow rates and bioconcentrations (the unknowns) by solving a system of linear constraints that incorporate empirical bioaccumulation data. If constraints are not contradictory, there are generally multiple admissible values that can be chosen. In our case, we derive a statistically well-founded solution by taking the mean^1^ of a set of random solutions obtained with Monte-Carlo sampling.

**Figure 1:**
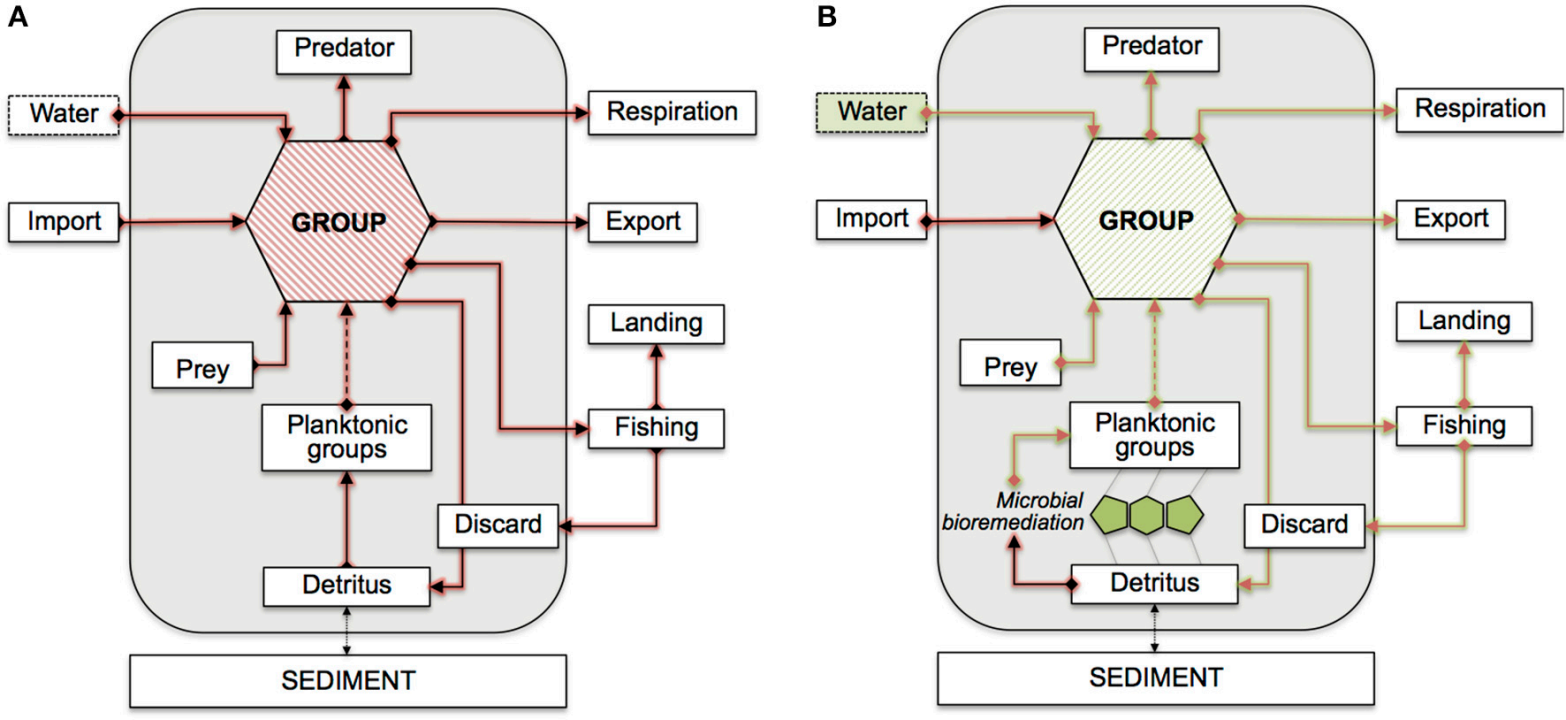
**Conceptual model of the Adriatic PCBs bioaccumulation network**. Flows are shown with respect to a generic functional group. Mass-balanced groups are enclosed in the gray boxes, externals are shown outside. The dashed arrow from planktonic groups indicate possible indirect connections. Feeding links from discard and detritus are omitted. **(A)** Red arrows indicate contaminant flows mediated by feeding connections. **(B)** Green arrows highlight the potential propagation of bioremediation effects. Possible bioremediation scenarios are assumed at the interface between detritus and planktonic groups (microbial loop), or in the water compartment.

Figure 1 illustrates the conceptual model and the topology of our PCBs bioaccumulation network; in Table 1, we provide a description of the contaminant flows and of the constrains used for their estimation. We consider the sum of PCBs congeners, expressed in ng g^−1^ wet weight-based. Biomasses are measured in t km^−2^ wet weight organic matter, and biomass flows in t km^−2^ year^−1^. PCBs flow rates are thus expressed in mg km^−2^ year^−1^. We denote the contaminant flow from prey *i* to predator *j* with *c*_*i*→*j*_, and the PCBs concentration in *i* with *C_i_*. We assume that biomass flows (*b*_*i*→*j*_) are known quantities and are estimated as reported in (Taffi et al., 2014).

**Table 1:** **Main flows in the PCBs bioaccumulation network and linear constraints for their estimation from data**.

**Mass balances:** ∑*_j_ c*_*j*→*i*_ − ∑*_j_ c*_*i*→*j*_ = 0
The bioconcentration of a generic group *i* is estimated under the mass-balance assumption; *j* ranges among groups and external compartments^a^.
**Concentration data:** *C*_*i*_ ⋈ *k*
where *k* is an input PCBs value used to constrain concentration *C_i_* and ⋈∈ {=,≤, ≥}. Note that an arbitrary number of data constraints can be included for the same group.
**Uptake from food/losses:** *c*_*j*→*i*_ = *b*_*j*→*i*_ · *C_j_*
The contaminant flow from group *j* to *i* is the product of the corresponding biomass flow *b*_*j*→*i*_ and the PCBs concentration in the source *j*. This equation characterizes both the contaminant uptake of predator *i* by consumption of prey *j* and the contaminant removal from *j* due to predation by *i*. If instead *i* is an external, the equation can express generic outflows to the export compartment (*c*_*j*→Export_); respiration flows (*c*_*j*→Respiration_), which account for part of the unassimilated fraction of ingested biomass; or removal due to fishing activity, which can be directed to the landings (*c*_*j*→Landing_) or to the discards (*c*_*j*→Discard_). The latter enters back the biomass cycle and is modeled as a mass-balanced group, with its own bioconcentration value.
**Uptake from generic imports:** *c*_Import→*i*_ = *b*_Import→*i*_ · *C*_*i*_
This class of constraints describes generic imports of PCBs coming from external contaminant inflows (e.g., immigration), which we group in the Import compartment. In this case, the PCBs concentration in the biomass imported into group *i* is assumed to be the same as in *i*.
**Uptake from environment:** *c*_Water→*i*_ = *w_i_* · *C*_Water_
where *w_i_* is the rate of contaminant uptake from water by group *i* and *C*_Water_ is the concentration in water^b^. Contaminant uptakes from water are not mediated by a biomass transfer and are estimated according to mass-balance constraints.
**Non-negativity of concentrations:** *C*_*i*_ ≥ 0

a *Natural detritus and planktonic groups are assumed to be in instant equilibrium with the water phase, and their concentration only depends on the concentration in water*.

b *When also *C*_Water_ is unknown, the constraint becomes non-linear and *w_i_* cannot be directly estimated. In this case, *c*_Water→*i*_ is treated as a single unknown. We assume null *w_i_* for compartments in rapid equilibrium with the water phase*.

### 2.2. Integration of PCBs degradation pathways into *P. putida* KT2440

**Figure 2:**
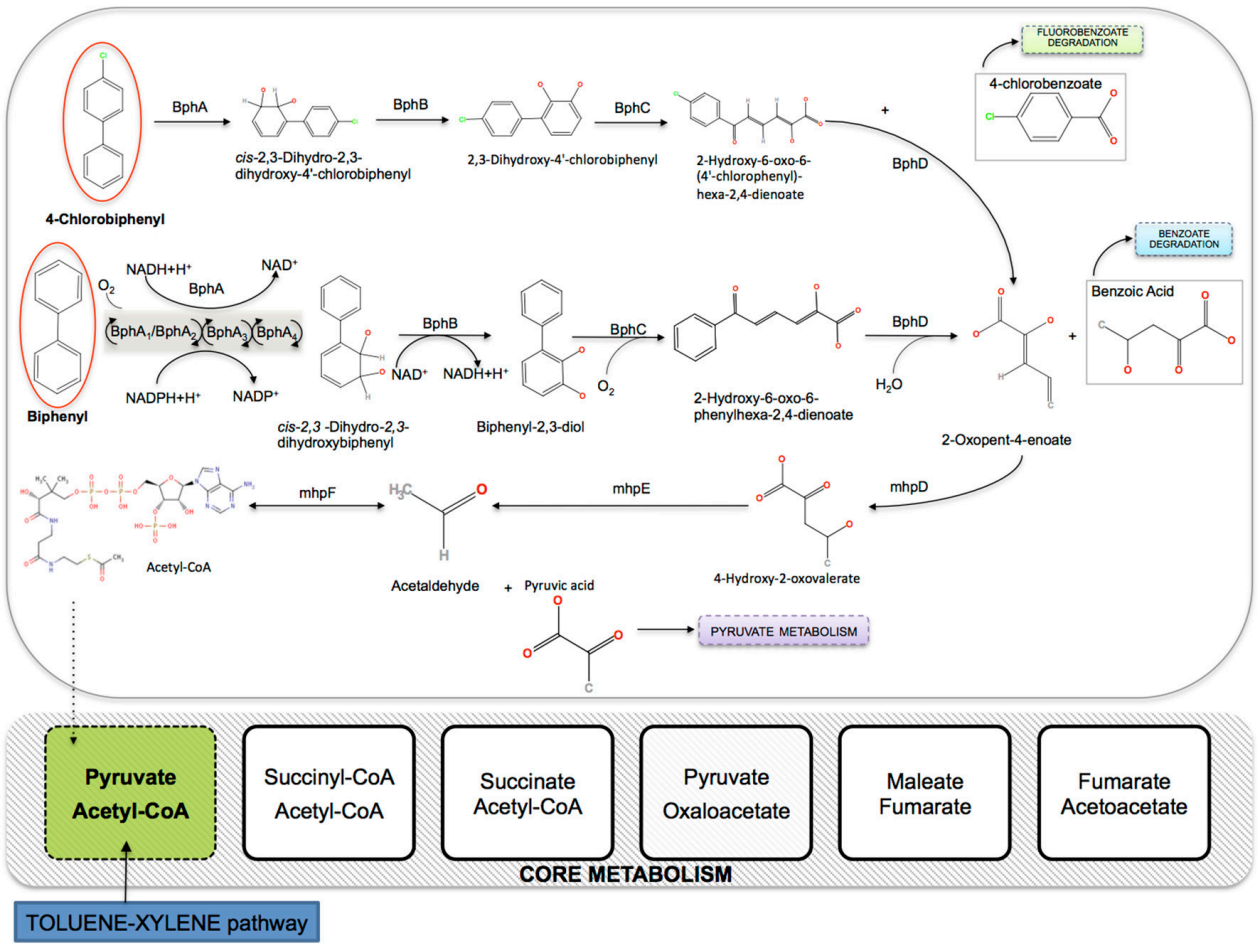
**Integration of the aerobic pathway of PCBs degradation in the core metabolism of *P. putida* KT2440 (iJN746)**. BphA, biphenyl 2,3-dioxygenase (multicomponent Rieske non-heme iron oxygenases); BphB, cis-2,3-dihydrobiphenyl-2,3-diol dehydrogenase; BphC, biphenyl-2,3-diol 1,2-dioxygenase; BphD, 2,6-dioxo-6-phenylhexa-3-enoate hydrolase; mhpD, 2-keto-4-pentenoate hydratase; mhpE, 4-hydroxy 2-oxovalerate aldolase; mhpF, acetaldehyde dehydrogenase.

Various environmental and biological factors limit the natural PCBs degradation process, among which the high selectivity of bacteria for specific PCBs congeners. Higher chlorinated congeners typically tend to accumulate in marine sediments, where anaerobic bacteria use these compounds by reductive dechlorination as alternative electron acceptors in their respiration processes, thus making PCBs less chlorinated and more aerobically degradable. This step is generally slow but crucial in the whole detoxification process, and various PCBs-dechlorinating bacteria, mainly belonging to the phylum *Chloroflexi*, have been isolated and characterized in different contaminated sites (Fava et al., 2003). The bioconversion process of less chlorinated PCBs congeners is performed by aerobic bacteria able to oxidatively cometabolize PCBs as the unique carbon source, since they encode biphenyl-metabolic enzymes *(bph)*. In order to have an effective degradation process, this aerobic step should ideally take place sequentially to the anaerobic step in the full microbial degradation pathway. As illustrated in Figure 2, the established aerobic route of PCBs elimination involves a set of enzymatic reactions acting on (chloro)biphenyl congeners to yield first benzoic acid, and then pyruvate and acetyl-CoA, that directly enter the Krebs cycle and increase the microbial biomass. Several aerobic bacteria are environmentally widely present and characterized as belonging to a variety of genera, including *Pseudomonas putida* (Furukawa, 2000). In particular, strains of *P. putida* have been isolated in water habitats and marine sediments (Garcia-Valdes et al., 1988).

In this work, we construct a synthetic model of PCBs degrading bacteria using the FBA approach (see Section 2.3), by extending the metabolic reconstruction of *P. putida* KT2440 (iJN746) in Nogales et al. (2008) with the aerobic degradation pathway of PCBs (KEGG pathway: map00621). As explained above, the pathway connects to the core metabolism of *P. putida* at the starting point of the citrate cycle (see Figure 2). The *P. putida* TOL-plasmid has been extensively used as a discovery platform for bioremediation purposes, since it encodes enzymes required for aromatic hydrocarbons degradation (e.g., toluene, benzoate, phenylacetate, nicotinate). Several studies report the genetic plasticity of different strains of *Pseudomonas* spp., showing the correspondence between gene clusters involved in biphenyl degradation pathways (Furukawa and Miyazaki, 1986) and genes for toluene degradation (Furukawa et al., 1993).

### 2.3. Bilevel flux balance analysis

Starting from biochemical reactions and stoichiometric coefficients, the Flux Balance Analysis (FBA) framework is based on the assumption of a metabolic steady state (Orth et al., 2010). That is, for each metabolite in the network, a balance is kept between the fluxes of those reaction in which the metabolite is a reactant, and those in which it is a product. Due to its ability to handle large biochemical networks without requiring kinetic parameters, FBA allows an effective *in silico* analysis of the invariant characteristics of the metabolic network at a low computational cost.

Formally, let *X_h_*, *h* = 1, …, *m* be the concentration of the *h*th metabolite in the network, and *v_k_*, *k* = 1, …, *n* be the flux of the *k*th reaction. Every *X_h_* must satisfy $\frac{dX_{h}}{dt}=\sum_{k\;=\;1}^{n}S_{hk}v_{k}$, where *S_hk_* is the stoichiometric coefficient of *h* in the *k*th reaction, such that *S_hk_* < 0 for substrates and *S_hk_* > 0 for products. Under the assumption of steady state conditions $\left(\frac{dX_{h}}{dt}=0\right)$, the flux balance constraint is *Sv* = 0.

Typically, there are more reactions than metabolites, thus equation *Sv* = 0 is a highly underdetermined linear system, leading to a plurality of solutions. The solution space can be restricted by imposing additional capacity constraints on the fluxes, e.g., defining the lower and upper bounds of each flux *V*^⊥^_*k*_ ≤ *v_k_* ≤ *V*^⊤^_*k*_, where *V*^⊥^_*k*_ and *V*^⊤^_*k*_ are the minimum and maximum flux rates for the *k*th reaction. A solution is taken through the maximization or the minimization of an objective function *Z* = ∑^*n*^_*k* = 1_ *f_k_v_k_*, where *f_k_* is the weight of the *k*th reaction. Under the above constraints, we obtain a convex optimization problem that can be efficiently solved with linear programming techniques.

When two objective functions are taken into account, an FBA problem can be formulated as a bilevel linear programming problem (e.g., for optimizing growth and product yield Burgard et al., 2003). This approach has been also adopted in metabolic engineering when optimizing models toward the overproduction of two metabolites simultaneously (Angione et al., 2013). Specifically, the FBA maximization problem becomes the *inner problem*, while an additional maximization problem constitutes the *outer problem*. The constraints of the outer maximization problem are the same as those of the inner problem, plus an additional constraint ensuring that the solution space is restricted to the solution of the inner problem. Formally, a bilevel maximization problem is defined as:

(1) $$\begin{array}{l} \text{max }g^{⊺}v\\ \text{subject to max }f^{⊺}v\;\\ \text{ subject to }Sv=0\\ \;V_{k}^{⊥}\leq v_{k}\leq V_{k}^{⊤} \end{array}$$

where *f* and *g* are vectors used to select the objectives. For instance, if in a two-objective problem we maximize the flux rates of the natural objective *v*_*k*_1__ (e.g., biomass production) and the synthetic objective *v*_*k*_2__ (e.g., contaminant uptake), we set *f*_*k*_1__ = 1 and *g*_*k*_2__ = 1. The solution of the bilevel problem (1) is a pair indicating the maximum natural objective (inner problem) allowed by the constraints *Sv* = 0 and *V*^⊥^_*k*_ ≤ *v_k_* ≤ *V*^⊤^_*k*_, and the maximum synthetic objective allowed in the flux distribution that maximizes the natural objective. The bilevel problem can be converted to a single-level problem using the duality theory applied to the inner problem, which is replaced by additional constraints for the outer problem.

### 2.4. FBA encoding of food web and integration with degradation pathways

We introduce a method for integrating the PCBs bioaccumulation network with the FBA-based metabolic reconstruction of *P. putida*. In the following, we use the more compact notation of chemical reactions to describe our FBA encoding, omitting the translation to the matrix form given in Section 2.3.

#### 2.4.1. FBA encoding

The basic idea is encoding each link *i* → *j* in the food web with a unary irreversible reaction with substrate *i* (the prey) and product *j* (the predator). Ecological compartments are thus translated into metabolites. Specifically, we derive the following set of reactions:

$$R_{FW}=\{(i,j):i\to_{\left[0,c_{i\to j}\right]}j\;|\;c_{i\to j}>0\}$$

where the rate of a reaction (*i*, *j*) (denoted by *r*_*i,j*_) is upper bounded by the original corresponding flow rate *c*_*i*→*j*_. This formulation admits a space of solutions with potentially reduced (even zeroed) contaminant flows, which is required in order to reproduce the contaminant removal by the bacterial metabolism.

Any admissible vector of fluxes for the reactions in *R_FW_* entails a food web with contaminant flows given by *r*_*i,j*_ for any group *i* and *j*. A reaction *i* → *j* having null flux indicates that prey *i* does not contribute to the contaminant uptake of predator *j*, e.g., when biomass transfer occurs between *i* and *j* (*b*_*i*→*j*_ > 0) but *i* has null contaminant concentration (*C_i_* = 0).

Additionally, we consider the following set of exchange reactions for expressing the external inputs and outputs of the food web:

$$\begin{array}{l} E_{FW}=\;\{e\to_{\left[0,+\;\infty \right)}\emptyset \;|\;e\in \{\text{Respiration},\text{ Export},\text{ Landing}\}\}\text{ and}\\ \;I_{FW}=\;\{\emptyset \to_{r}i\;|\;i\in \{\text{Water},\text{ Import}\}\text{ and }r\;=\;\sum_{j}c_{i\to j}\} \end{array}$$

The set *E_FW_* contains, for each external sink *e* of the food web, an unbounded export reaction from *e*. Similarly, the set of import reactions *I_FW_* has an uptake reaction for each external source, but in this case the uptake rate is set to the sum of all flows imported through *i* in the contaminated network (∑*_j_ c*_*i*→*j*_). Note that it is sufficient to constrain the import reactions in order to obtain a consistent FBA encoding of the bioaccumulation model. Indeed, by mass-balance, throughflow values are conserved by the encoding and it can be shown that the food webs entailed by the reactions in *R_FW_* ∪ *E_FW_* ∪ *I_FW_* are all identical to the original network, up to redistribution of external exports.

Finally, for a generic group *i*, the resulting bioconcentration *C_i_* in the entailed network is computed as the ratio between the total contaminant outflows (as reaction fluxes) and the total biomass outflows:

$$C_{i}=\frac{\sum_{j}r_{i,j}}{\sum_{j}b_{i\to j}}.$$

#### 2.4.2. Integration with *P. putida* metabolism

An effective way to accomplish this task is adding a dummy metabolite *x*, which serves as the interface between the encoded bioaccumulation model and the bacterial reactions. In particular, *x* represents the unbounded sink for all the food web groups we aim to remediate; and the unbounded source for all the metabolites describing PCBs molecules in the *P. putida* metabolism (in our case, Biphenyl and 4-Chlorobiphenyl). Clearly, these *interface reactions* could also be bounded with arbitrary or experimentally measured limiting factors to bioremediation efficiency, as done in Section 3 for evaluating different degrees of bioremediation.

We define the *bioremediation problem*, as that of *maximizing the amount of remediated flow*, i.e., the sum of fluxes exiting^2^ metabolite *x* in the integrated metabolism-food web network. Formally,

$$\begin{array}{l} \text{ max }\sum_{\bar{x}\to x^{\prime }\in I}r_{\bar{x},x^{\prime }}\\ \text{subject to reactions }R_{FW}\cup E_{FW}\cup I_{FW}\cup I\cup R \end{array}$$

where *I* denotes the set of interface reactions; *R* is the set of reactions in the *P. putida* metabolism; and *R_FW_*, *E_FW_* and *I_FW_* describe the encoded food web.

Evidently, not all integrations are ecologically and biologically plausible. In our model, we consider two bioremediation scenarios, as also shown in Figure 1:

- *Scenario 1: Bioremediation of detritus groups*. This hypothesis is based on the fact that the microbial loop, where bioremediating bacteria are assumed to naturally operate, is located at the interface between natural detritus and planktonic groups (both included in our food web). Thus, we redirect the outflows from detritus to the microbial metabolism. The same applies to the discard group, treated as a detritus in our model. The integration reactions are:
  $$\begin{array}{l} I_{1}\;=\;\{\text{Detritus }\to_{\left[0,+\;\infty \right)}\bar{x},\text{ Discard }\to_{\left[0,+\;\infty \right)}\;\bar{x},\;\bar{x}\;\to_{\left[0,+\;\infty \right)}\\ \text{ Biphenyl},\;\bar{x}\;\to_{\left[0,+\;\infty \right)}\text{4-Chlorobiphenyl}\} \end{array}$$
- *Scenario 2: Bioremediation of water compartment*. This case describes the effects of an *in situ bioremediation* process of PCBs, regarded as acting within the water compartment (an external in our model), decreasing PCBs concentrations in the whole surrounding environmental media. The integration reactions are:
  $$\begin{array}{l} I_{2}\;=\;\{\text{Water}\to_{\left[0,+\;\infty \right)}\bar{x},\;\bar{x}\to_{\left[0,+\;\infty \right)}\text{ Biphenyl},\;\bar{x}\;\to_{\left[0,+\;\infty \right)}\\ \text{ 4-Chlorobiphenyl}\} \end{array}$$

The integrated models have been obtained after converting PCBs flows (mg km^−2^ year^−1^) to the flux units used in FBA (mmol h^−1^ gDW^−1^). The conversion factor is *k* = 1/ (*m* · *t* · *n*), where *m* is the molar mass of a PCBs molecule (Biphenyl: 154.2078 mol g^−1^; 4-Chlorobiphenyl: 188.6529 mol g^−1^); *t* = 8760 h is the number of hours per year; and *n* is the amount (gDW) of actively remediating *P. putida* in the unit of space (1 km^2^). *k* can be applied to all the PCBs flows, or as a stoichiometric coefficient in the interface reactions. In our model, we set *n* = 10^−3^ gDW, enough to import the totality of the connected PCBs flows into the *P. putida* metabolism, and to avoid numerical errors in the optimization procedure due to excessively small flux values. However, marine metagenomic data can be used to have a finer estimation of parameter *n*.

### 2.5. Ecological network indices

In order to assess the effects of bioremediation on our contaminated food web, we combine the evaluation of bioconcentrations with the study of ecological network indices. Typically, global indices (Kones et al., 2009) are used to derive unique descriptors of the structure and properties of the whole ecosystem. On the other hand, indices of species centrality (Jordán, 2009) are typically employed for conservation purposes and give a measure of species importance in the global functioning of the ecosystem. These notions can be naturally applied to the study of our contaminated ecological network, where central species are those having a crucial role in the trophic diffusion of PCBs among other species, while global indices provide insights into the degree of ecosystem pollution. In our evaluation, we consider *Flow Betweenness Centrality* (FBC) (Freeman et al., 1991) and *Link Density*, (LD) even if our framework can be applied to the study of arbitrary network indices.

FBC gives the topological importance of a species in maintaining the flows among other groups. The FBC of a group *i*, *FBC_i_*, is defined as

$$FBC_{i}=\sum_{j\;\neq \;k,j\;\neq \;i,k\;\neq \;i}\left(max_{G}\;c_{j\to k}-max_{G\setminus i}\;c_{j\to k}\right)$$

where *max_G_ c*_*j*→*k*_ is the maximum flow between *j* and *k* in the considered food web *G* and *max*_*G*\*i*_ *c*_*j*→*k*_ is the maximum flow between *j* and *k* in the same network without group *i*.

We employ *LD* to obtain a structural and qualitative descriptor of the network. It expresses the average number of active links (with non-null flow) per species and, ideally, from an effective bioremediation strategy, we expect a substantial breakdown of this property. It is calculated as:

$$LD=\frac{\sum_{i}\sum_{j}\left(c_{i\to j}>0\right)}{n}$$

where *n* is the number of groups in the network.

## 3. Results

The approach for the estimation of the PCBs bioaccumulation model and for the analysis of network indices was implemented in R (using packages *LIM* van Oevelen et al., 2010 and *sna* Butts, 2008). The MATLAB-based *COBRA toolbox* (Schellenberger et al., 2011) was used for constructing and analyzing our extension of the *P. putida* metabolism as well as for the reaction-based encoding and integration of the food web. The extended *P. putida* model was deposited in BioModels Database (Li et al., 2010), id: MODEL1407250000. The code and the models developed in this work are available at http://www.nicolapaoletti.com/files/research/models/Frontiers_model.zip.

### 3.1. PCBs metabolism in KT2440 and interactions with toluene degradation

**Figure 3:**
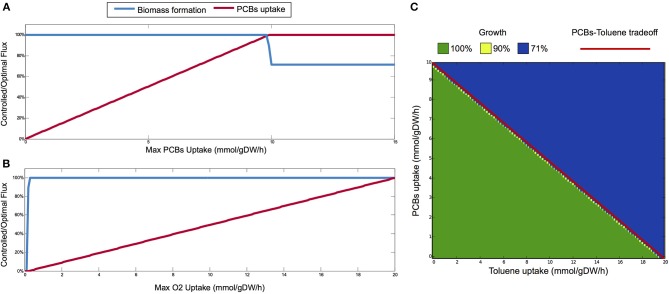
**(A)** Bilevel analysis on the *P. putida* metabolism: we study the optimal growth rates on the solution space of optimal PCBs uptake (L1), when the upper bound of the latter ranges from 0 to 15 mmol h^−1^ gDW^−1^. The maximum PCBs uptake rate is 10 mmol h^−1^ gDW^−1^, and the optimal growth rate is thus achieved for almost the whole range of PCBs uptake. **(B)** Single-level analysis: controlled/optimal flux of biomass and PCBs uptake rate at different oxygen levels, which in our case are determined also by different depths. The *P. putida* is able to keep a high growth rate also on low oxygen. The linear relationship between PCBs and oxygen uptake rates is in keeping with the fact that the uptake of PCBs depends on aerobic degradation. **(C)** Interdependence between toluene and PCBs uptake and corresponding phenotypic phase plane (PhPP). The red dashed line shows the trade-off between toluene and PCBs uptakes, obtained with a bilevel analysis of optimal toluene uptake (L2), over the configuration maximizing PCBs uptake (L1), by limiting the latter from 0 to 10 mmol h^−1^ gDW^−1^. The symmetric bilevel problem (with toluene limited from 0 to 20 mmol h^−1^ gDW^−1^) gives the same linear front. This tradeoff delineates two phenotypes in the PhPP analysis (L2: biomass, L1: toluene+PCBs uptakes): in the lower half (green region), we have optimal growth; in the upper half (blue region), growth is limited to 71% of the optimal growth.

By applying bilevel FBA, the growth rate of *P. putida* remains at the maximum value (1.3975 h^−1^) for PCBs uptake rate up to 9.8 mmol h^−1^ gDW^−1^ (Figure 3A). The maximum PCBs uptake supported by *P. putida* is registered at 10 mmol h^−1^ gDW^−1^, since the rate of PCBs uptake stays constant for upper bounds greater than this value. Therefore, optimal growth is maintained until the rate of PCBs uptake is almost at its maximum, while for uptake rates greater than 9.8 mmol h^−1^ gDW^−1^, biomass production drops to 71% of its optimal value (1 h^−1^). Further, the addition of the PCBs bioremediation pathways to the *P. putida* metabolism does not result in an increased growth rate.

In order to investigate the relationship between growth rate and oxygen uptake, and between PCBs uptake and oxygen uptake, we apply a single-level FBA analysis. In Figure 3B, we evaluate the optimal flux of biomass and PCBs uptake rate at different levels of oxygen uptake (simulating different depths in the marine environment). While the optimal PCBs uptake rate is linear with the maximum oxygen uptake rate allowed, the growth rate increases quickly for low import of oxygen until 0.4 mmol h^−1^ gDW^−1^, and then remains stable even at high oxygen uptake. The *P. putida* is able to keep a high growth rate also with low oxygen, which reproduces the environmental conditions describing the proposed first bioremediation scenario. The linear relationship between PCBs and oxygen uptake rates is in keeping with the fact that the uptake of PCBs depends on the aerobic degradation pathway.

We also analyse the interdependence between the PCBs degradation pathways (introduced in this work) and that of toluene (in the original reconstruction). We derive an optimality front between them by solving two bilevel problems. In the first, we evaluate the maximum toluene uptake when PCBs imports are favored, while in the second problem, we consider the symmetric objectives. Both problems identify the identical linear trade-off (red dashed line in Figure 3C), evidencing that *P. putida* is not able to optimally support multiple degradation pathways. We further perform a phenotypic phase plane (PhPP) analysis by coupling the biomass production objective with varying PCBs and toluene uptake rates. In this case, we seek to optimize growth on top of the configuration maximizing both degradation pathways (the sum of PCBs and toluene uptakes). The PCBs-toluene tradeoff delineates two regions in the phenotypic space: when the uptakes of PCBs and toluene are below the optimal front, maximum growth is achieved (100%, green area in Figure 3C); and when they exceed the front, we found a reduced growth (71%, blue area). Negligible regions with 90% growth (yellow points) are found at the border between the two phenotypes. Specifically, we observe that optimal growth is achieved for PCBs and toluene fluxes strictly below this trade-off, implying that reduced growth occurs also at high uptake values (PCBs flux > 9.8 mmol h^−1^ gDW^−1^, toluene flux > 19.7 mmol h^−1^ gDW^−1^), as also seen for the PCBs case in Figure 3A. It follows that, apart from extreme uptake values, the extended metabolic network of *P. putida* robustly gives optimal growth yields even in the strain designs targeted to the maximization of multiple degradation pathways.

### 3.2. Bioremediation effects on bioaccumulation and species centrality

We analyse the integrated models obtained by applying the two scenarios introduced in Section 2.4. In Scenario 1, microbial degradation pathways reduce contaminant concentrations through outflows from natural detritus and fishing discards (functional groups 38 and 39, trophic level=1), simulating a bioremediation at the level of the microbial loop. In Scenario 2, PCBs bioremediation is assumed to act in the water compartment by reducing simultaneously all the PCBs uptakes in each functional group. The following results are obtained by solving the bioremediation problem (Section 2.4) and computing bioconcentrations and network indices on the resulting (the entailed) bioaccumulation networks.

**Figure 4:**
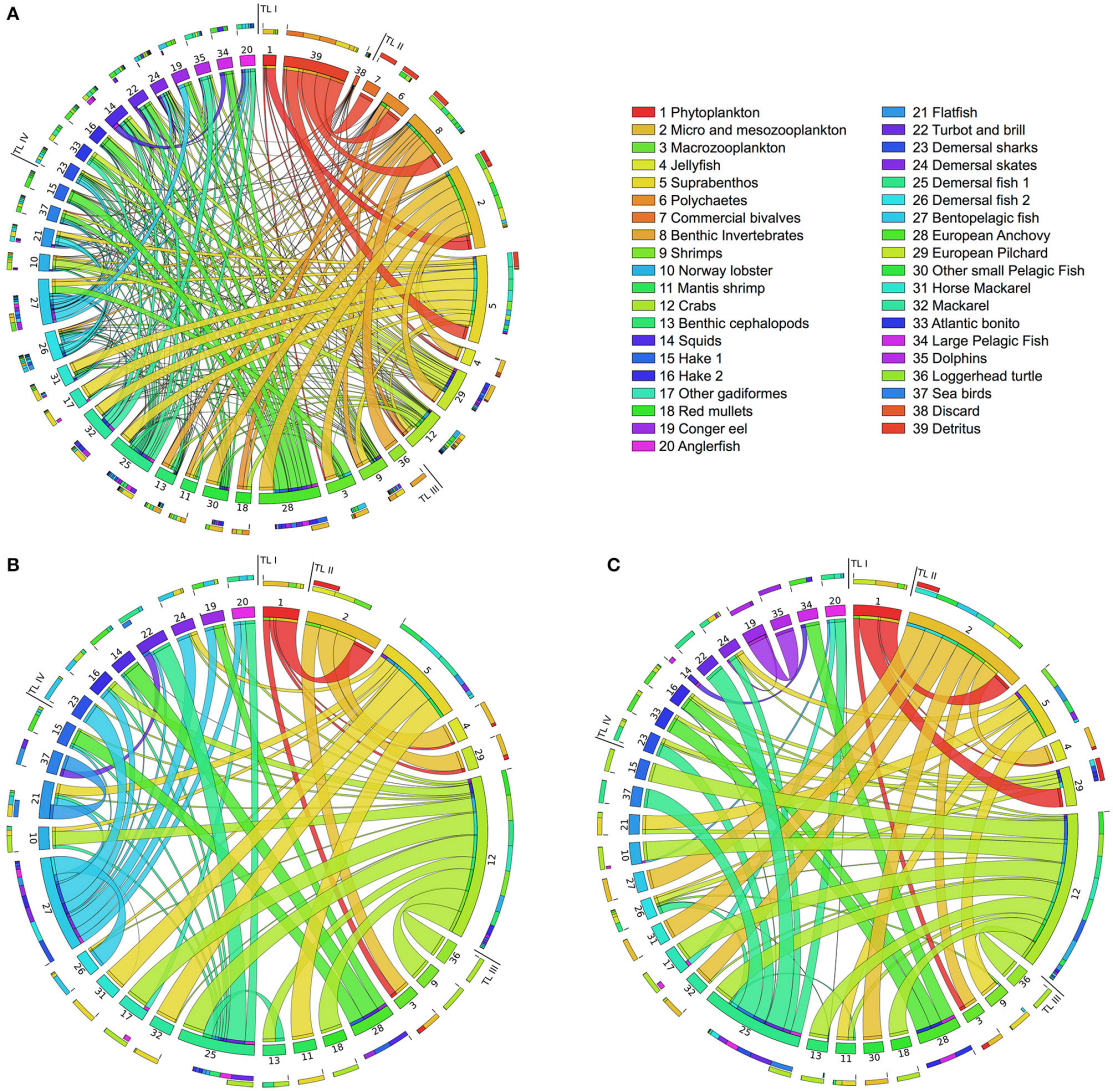
**Circular plot of the Adriatic food web in the three cases considered: PCBs bioaccumulation network without bioremediation (A; Scenario 0); at maximum bioremediation efficiency for the natural bioremediation acting on detritus and discard (B; Scenario 1); and the in-situ bioremediation acting on the water compartment (C; Scenario 2)**. Functional groups are located clock-wise in ascending trophic level order. Ribbons represent feeding links carrying PCBs flows. Each ribbon takes the same color as its source node (the prey), and thickness is proportional to the contribution of the source in the diet of the target node (the predator). In each group, the outmost stacked bars summarize its diet composition and its contribution to predators' diet. External and flows to detritus groups are not displayed. The top-right table lists the functional groups of the Adriatic food web and their ID numbers. Images has been obtained by using the *Circos* tool (Krzywinski et al., 2009).

In Figure 4, we illustrate in a circular layout the PCBs bioaccumulation networks of a business-as-usual case without bioremediation, hereafter called *Scenario 0* [plot (a)], and of the above two scenarios when no limits to the bioremediation efficiency are imposed [plot (b, c)]. Figure 4 depicts only the contaminant flows mediated by feeding links. Plot (a) highlights that in Scenario 0, contaminant diffusion throughout the food web is driven by a dense network of trophic connections, each of them carrying a non-null PCBs flow.

In Scenario 1 [plot (b)], we clearly notice a simpler pattern of PCBs contamination among functional groups, due to a considerable reduction of feeding links active in the transport of PCBs. Specifically, the redirection of outflows from detritus and discard out of the food web causes the inactivation of several PCBs flows, and subsequent presence of groups with null PCBs concentration. Therefore, these groups (not plotted) are disconnected from the bioaccumulation network but still active in the biomass network. They include detritus (38) and discard (39); detritivores (6,7,8); group 30, which only feed on planktonic groups; and groups feeding on those mentioned so far (33, 34, 35). Other variations are detectable in groups 5, 27, and 28 (detritivores and planktivores) that no longer acquire PCBs from food. Finally, we can observe that groups 12 and 27 gain in this scenario a central role in the contaminant diffusion, becoming the preferential source of most of their predators, while in Scenario 0 their contribution appears less relevant.

The bioaccumulation network under Scenario 2 [plot (c)] exhibits a similar structure to Scenario 1. Detritus groups (38,39) and detritivores (6,7,8) are no longer connected to the rest of the food web, showing that bioremediation of the water compartment tends to disrupt the pathways of contaminant uptake at the lowest trophic levels. Another similarity is the promotion of group 12 as a central node in the acquisition of PCBs by its predators. On the other hand, group 27 has no outgoing PCBs flows, while in plot (b) the opposite situation (no inflows) is observed for the same group. In general, we notice a lower number of active links with respect to Scenario 1, especially in species at higher trophic levels.

**Figure 5:**
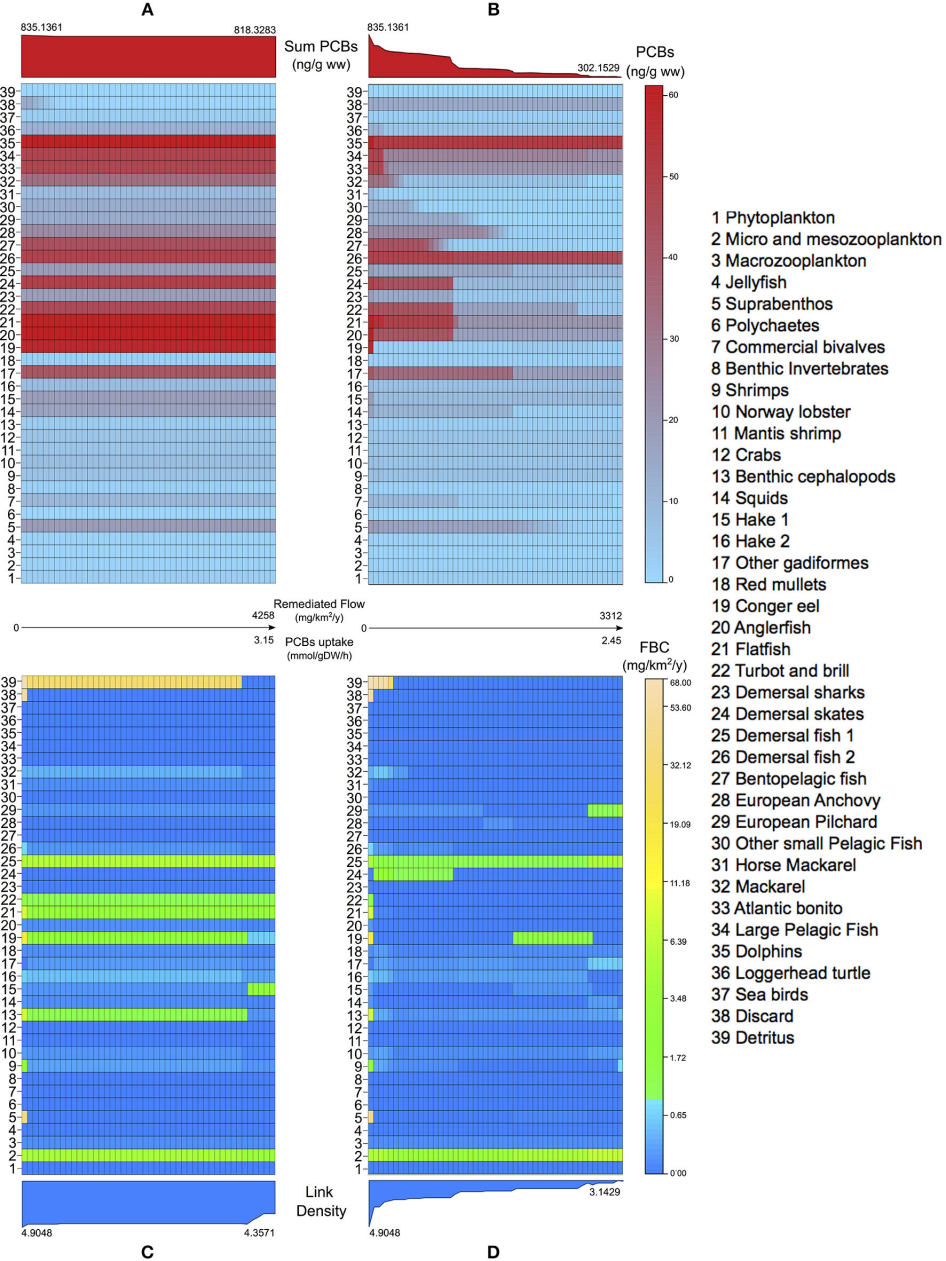
**Levelplots of PCBs concentrations (A,B) and flow betweenness centralities (C,D) in Adriatic species (y-axis) at increasing amounts of contaminant removed by bacterial uptake (x-axis) in the natural (A,C) and *in situ* (B,D) bioremediation scenarios**. In the middle, the final amount of remediated flow and the corresponding PCBs uptake are reported for the two scenarios. Plots on the top of **(A,B)** show the evolution of the sum of PCBs in the food web at increasing degrees of bioremediation. Plots on the bottom of **(C,D)** show the effects of bioremediation in the link density of the bioaccumulation network.

Another kind of analysis enabled by our framework is the study of the networks obtained by solving the bioremediation problem at increasing efficiencies, limiting the amount of PCBs flow allowed into the bacterial metabolism. In both scenarios, we analyse the variations in PCBs bioconcentrations (Figures 5A,B) and in the topological importance of functional groups, measured with the FBC index introduced in Section 2.5 (Figures 5C,D). We report a difference between the maximum remediated PCBs flows in the two scenarios (4258 mg km^−2^ year^−1^ and 3312 mg km^−2^ year^−1^, respectively), which mainly depends on the structure of the network. Applying the conversion factor in Section 2.4, the maximum remediated flow in Scenario 1 corresponds to a PCBs uptake rate of 3.15 mmol h^−1^ gDW^−1^ (31.5% of the maximum uptake), while in Scenario 2 to 2.45 mmol h^−1^ gDW^−1^ (24.5% of the maximum uptake).

As regards Scenario 1, no remarkable reductions in bioconcentrations are observed in the entire food web [see plot (a)], apart from the natural detritus and the discard groups, whose PCBs values are zeroed at 89 and 16%, respectively, of the maximum bioremediation efficiency. We register only minor drops in a number of groups at TL 4 (14, 16, 23, 24) and in group 37 (feeding on discard). This tendency is also visible in the sum of PCBs, which is practically constant.

On the other hand, Scenario 2 gives a considerable decrease in the bioconcentrations of all groups [plot (b)]. This is explained by the fact that the estimated uptakes from water constitute an important fraction of imported contaminant, whose degradation also mitigates, indirectly, uptakes from food. The only exceptions are natural detritus and discard (38, 39), which have null PCBs flow from water (see Table 1), and groups 26 and 35 where, according to our estimation, water imports are the least relevant external uptakes. In general, the sum of PCBs concentrations shows a constant and gradual decreasing trend, even though steeper reductions are observable at low values of microbial degradation (2% of maximum efficiency gives a 17% drop in the sum of PCBs), and at about 34% of the maximum bioremediation (leading to a 48% reduction of the initial total PCBs).

In Scenario 1, the analysis of the FBC index [plot (c)] highlights the topological importance of natural detritus in the bioaccumulation network, which derives from the fact that every group in the food web contributes (via natural death and unassimilated food) to its contaminant uptake. Indeed, this group maintains its central role up to 89% of bioremediation efficiency. After this point, a structural disruption occurs, related to the detritus becoming disconnected from the network (i.e., no incident flows). This leads to cascade effects also in the centrality of groups 13, 15, 16, 19, and 32. Apart from this case, FBC exhibits quite a robust pattern, showing a number of groups (2, 21, 22, 25) with unchanged centralities regardless of the amount of bioremediated flux. This structural robustness is evidenced also by the link density values, indicating that, globally, the number of links active in the PCBs diffusion is relatively constant.

On the contrary, Scenario 2 [plot (d)] produces prominent changes in the centrality of most species. Here, natural detritus loses its dominant role in the network at 10% of maximum bioremediation. Moreover, at 34% of efficiency, we observe a sudden fall in the FBC of group 24, as also registered on its bioconcentration values [see plot (b)]. Only functional groups 2 and 25 show robust topological importance, in agreement with Scenario 1. The evolution of the link density index also evidences the high sensitivity of the network structure. Indeed, the index reaches an average of 3.1429 active links per group, 36% lower than the initial value.

## 4. Discussion

Recent biotechnological advances and novel discovery tools in marine metagenomics are paving the way for new integrated solutions in environmental bioengineering, turning empirical hypotheses into practical methods. In this context, we presented a computational framework for the analysis of contaminated ecosystems and for the evaluation of different hypothetical bioremediation scenarios. We considered the case of PCBs bioaccumulation in the Adriatic food web and PCBs degradation mediated by *Pseudomonas putida*. Our framework is based on a range of multi-scale analyses obtained by combining well-established methods in ecological modeling (Linear Inverse Modeling and Ecological Network Analysis) and Systems Biology (Flux Balance Analysis). We showed how to derive optimal remediation strategies that yield the highest decrease of bioaccumulation phenomena in species. In addition, more realistic scenarios can be reproduced that take into account environmental limiting factors influencing the potential of natural or synthetically designed microbial pathways.

Our computational experiments indicated that the extended *P. putida* metabolic model supports well the degradation of PCBs, and that a substantial drop of PCBs concentration in Adriatic species is achieved with comprehensive bioremediation strategies (e.g., Scenario 2: bioremediation of water compartments), while natural bioremediation (e.g., Scenario 1: bioremediation of detritus group) proved to be less effective. Results also highlight how remediation patterns vary among species in function of their feeding relationships. The study of ecological network indices allowed the evaluation of emerging global ecosystem properties under different bioremediation scenarios and degradation efficiencies.

To the best of our knowledge, this is the first computational method linking genome-scale reconstructions of bacterial metabolism with food web bioaccumulation models for designing and analysing bioremediation strategies. Approaches based on high dimensional omics data and network inference methods (Perkins et al., 2011; Williams et al., 2011) have been proposed for predicting the exposure of organisms to contaminated sites and for reconstructing adverse outcome pathways (Ankley et al., 2010). From the experimental side, Kupryianchyk et al. (2013) were the first to study how in situ sediment treatment reduces bioaccumulation at different trophic levels in aquatic food chains.

In this work we wanted to stress a different view on marine ecosystems, regarding them not just as ensembles of macro-species, but as complex multiscale networks linking classical food webs and microbial groups, toward a new perspective of “eco-metabolic” networks. We believe that bringing the study of microbial metabolic activity into the field of ecotoxicological modeling can highlight bottlenecks and advantages of different bioremediation approaches and shed light on the ecological role of marine microbial life. Furthermore, PCBs degrading bacteria live in communities structurally organized in biofilms (Abraham et al., 2002), where genetic events like recombination, conjugation and gene transfer (Dahlberg et al., 1998) can naturally lead to new metabolic pathways for pollutant degradation. In this perspective, our framework could be extended from single organism models to the bioengineering of bacterial consortia, e.g., following Brenner et al. (2008) and Klitgord and Segrè (2010), where natural genetic interactions can be explored and synthetically optimized for different persistent contaminants.

### Conflict of interest statement

The authors declare that the research was conducted in the absence of any commercial or financial relationships that could be construed as a potential conflict of interest.

## Footnotes

^1^Being a linear operation, the mean of valid solutions to a system of linear constraints is in turn a valid solution to the system (see also van Oevelen et al., 2010).

^2^Due to the mass balance assumption of FBA, influxes could have been equivalently considered.

## References

[B1] Abraham W.-R. Nogales B. Golyshin P. N. Pieper D. H. Timmis K. N. (2002). Polychlorinated biphenyl-degrading microbial communities in soils and sediments. Curr. Opin. Microbiol. 5, 246–253 10.1016/S1369-5274(02)00323-5 12057677

[B2] Angione C. Carapezza G. Costanza J. Liò P. Nicosia G. (2013). Pareto optimality in organelle energy metabolism analysis. IEEE/ACM Trans. Comput. Biol. Bioinf. 10, 1032–1044 10.1109/TCBB.2013.95 24334395

[B3] Ankley G. T. Bennett R. S. Erickson R. J. Hoff D. J. Hornung M. W. Johnson R. D. (2010). Adverse outcome pathways: a conceptual framework to support ecotoxicology research and risk assessment. Environ. Toxicol. Chem. 29, 730–741 10.1002/etc.34 20821501

[B4] Bayarri S. Baldassarri L. T. Iacovella N. Ferrara F. Domenico A. d. (2001). Pcdds, pcdfs, pcbs and dde in edible marine species from the adriatic sea. Chemosphere 43, 601–610 10.1016/S0045-6535(00)00412-4 11372844

[B5] Bedard D. L. Quensen J. F. III (1995). Microbial reductive dechlorination of polychlorinated biphenyls, in Microbial Transformation and Degradation of Toxic Organic Chemicals, eds Young L. Y. Cerniglia C. E. (New York, NY: Wiley-Liss Division, John Wiley & Sons, Inc.), 127–216

[B6] Brenner K. You L. Arnold F. H. (2008). Engineering microbial consortia: a new frontier in synthetic biology. Trends Biotechnol. 26, 483–489 10.1016/j.tibtech.2008.05.004 18675483

[B7] Brown J. F. Jr. Bedard D. L. Brennan M. J. Carnahan J. C. Feng H. Wagner R. E. (1987). Polychlorinated biphenyl dechlorination in aquatic sediments. Science 236, 709–712 10.1126/science.236.4802.709 17748310

[B8] Burgard A. P. Pharkya P. Maranas C. D. (2003). Optknock: a bilevel programming framework for identifying gene knockout strategies for microbial strain optimization. Biotechnol. Bioeng. 84, 647–657 10.1002/bit.10803 14595777

[B9] Butts C. T. (2008). Social network analysis with sna. J. Stat. Softw. 24, 1–51 18618019 10.18637/jss.v024.i01 PMC2447931

[B10] Christensen V. Walters C. (2004). Ecopath with Ecosim: methods, capabilities and limitations. Ecol. Model. 172, 109–139 10.1016/j.ecolmodel.2003.09.003

[B11] Coll M. Piroddi C. Steenbeek J. Kaschner K. Lasram F. B. R. Aguzzi J. (2010). The biodiversity of the mediterranean sea: estimates, patterns, and threats. PLoS ONE 5:e11842 10.1371/journal.pone.0011842 20689844 PMC2914016

[B12] Coll M. Santojanni A. Palomera I. Tudela S. Arneri E. (2007). An ecological model of the northern and central adriatic sea: analysis of ecosystem structure and fishing impacts. J. Marine Syst. 67, 119–154 10.1016/j.jmarsys.2006.10.002

[B13] Corsolini S. Aurigi S. Focardi S. (2000). Presence of polychlorobiphenyls (pcbs) and coplanar congeners in the tissues of the mediterranean loggerhead turtle caretta caretta. Marine Pollut. Bull. 40, 952–960 10.1016/S0025-326X(00)00038-2

[B14] Dahlberg C. Bergström M. Hermansson M. (1998). In situ detection of high levels of horizontal plasmid transfer in marine bacterial communities. Appl. Environ. Microbiol. 64, 2670–2675 9647846 10.1128/aem.64.7.2670-2675.1998 PMC106442

[B15] Danovaro R. Corinaldesi C. D'Onghia G. Galil B. Gambi C. Gooday A. J. (2010). Deep-sea biodiversity in the mediterranean sea: the known, the unknown, and the unknowable. PLoS ONE 5:e11832 10.1371/journal.pone.0011832 20689848 PMC2914020

[B16] Fava F. Zanaroli G. Young L. (2003). Microbial reductive dechlorination of pre-existing pcbs and spiked 2, 3, 4, 5, 6-pentachlorobiphenyl in anaerobic slurries of a contaminated sediment of venice lagoon (italy). FEMS Microbiol. Ecol. 44, 309–318 10.1016/S0168-6496(03)00069-2 19719612

[B17] Fenchel T. (2008). The microbial loop–25 years later. J. Exp. Marine Biol. Ecol. 366, 99–103 10.1016/j.jembe.2008.07.013

[B18] Freeman L. C. Borgatti S. P. White D. R. (1991). Centrality in valued graphs: a measure of betweenness based on network flow. Soc. Netw. 13, 141–154 10.1016/0378-8733(91)90017-N

[B19] Furukawa K. (2000). Biochemical and genetic bases of microbial degradation of polychlorinated biphenyls (pcbs). J. Gen. Appl. Microbiol. 46, 283–296 10.2323/jgam.46.283 12483570

[B20] Furukawa K. Hirose J. Suyama A. Zaiki T. Hayashida S. (1993). Gene components responsible for discrete substrate specificity in the metabolism of biphenyl (bph operon) and toluene (tod operon). J. Bacteriol. 175, 5224–5232 8349562 10.1128/jb.175.16.5224-5232.1993 PMC204990

[B21] Furukawa K. Miyazaki T. (1986). Cloning of a gene cluster encoding biphenyl and chlorobiphenyl degradation in pseudomonas pseudoalcaligenes. J. Bacteriol. 166, 392–398 3009395 10.1128/jb.166.2.392-398.1986 PMC214617

[B22] Garcia-Valdes E. Cozar E. Rotger R. Lalucat J. Ursing J. (1988). New naphthalene-degrading marine pseudomonas strains. Appl. Environ. Microbiol. 54, 2478–2485 3202629 10.1128/aem.54.10.2478-2485.1988 PMC204290

[B23] Hendriks A. J. van der Linde A. Cornelissen G. Sijm D. T. (2001). The power of size. 1. rate constants and equilibrium ratios for accumulation of organic substances related to octanol-water partition ratio and species weight. Environ. Toxicol. Chem. 20, 1399–1420 10.1002/etc.5620200703 11434281

[B24] Jordán F. (2009). Keystone species and food webs. Philos. Trans. R. Soc. B Biol. Sci. 364, 1733–1741 19451124 10.1098/rstb.2008.0335 PMC2685432

[B25] Klitgord N. Segrè D. (2010). Environments that induce synthetic microbial ecosystems. PLoS Comput. Biol. 6:e1001002 10.1371/journal.pcbi.1001002 21124952 PMC2987903

[B26] Kones J. K. Soetaert K. van Oevelen D. Owino J. O. (2009). Are network indices robust indicators of food web functioning? a monte carlo approach. Ecol. Model. 220, 370–382 10.1016/j.ecolmodel.2008.10.012

[B27] Krzywinski M. Schein J. Birol İ. Connors J. Gascoyne R. Horsman D. (2009). Circos: an information aesthetic for comparative genomics. Genome Res. 19, 1639–1645 10.1101/gr.092759.109 19541911 PMC2752132

[B28] Kupryianchyk D. Rakowska M. Roessink I. Reichman E. Grotenhuis J. Koelmans A. (2013). *In situ* treatment with activated carbon reduces bioaccumulation in aquatic food chains. Environ. Sci. Technol. 47, 4563–4571 10.1021/es305265x 23544454

[B29] Laender F. D. Oevelen D. V. Middelburg J. J. Soetaert K. (2009). Incorporating ecological data and associated uncertainty in bioaccumulation modeling: methodology development and case study. Environ. Sci. Technol. 43, 2620–2626 10.1021/es802812y 19452926

[B30] Li C. Donizelli M. Rodriguez N. Dharuri H. Endler L. Chelliah V. (2010). Biomodels database: an enhanced, curated and annotated resource for published quantitative kinetic models. BMC Syst. Biol. 4:92 10.1186/1752-0509-4-92 20587024 PMC2909940

[B31] Lovley D. R. (2003). Cleaning up with genomics: applying molecular biology to bioremediation. Nat. Rev. Microbiol. 1, 35–44 10.1038/nrmicro731 15040178

[B32] Marcotrigiano G. Storelli M. (2003). Heavy metal, polychlorinated biphenyl and organochlorine pesticide residues in marine organisms: risk evaluation for consumers. Vet. Res. Commun. 27, 183–195 10.1023/B:VERC.0000014137.02422.f4 14535387

[B33] Nogales J. Palsson B. Ø. Thiele I. (2008). A genome-scale metabolic reconstruction of *Pseudomonas putida* kt2440: ijn746 as a cell factory. BMC Syst. Biol. 2:79 10.1186/1752-0509-2-79 18793442 PMC2569920

[B34] Orth J. Thiele I. Palsson B. (2010). What is flux balance analysis? Nat. Biotechnol. 28, 245–248 10.1038/nbt.1614 20212490 PMC3108565

[B35] Perkins E. J. Chipman J. K. Edwards S. Habib T. Falciani F. Taylor R. (2011). Reverse engineering adverse outcome pathways. Environ. Toxicol. Chem. 30, 22–38 10.1002/etc.374 20963852

[B36] Perugini M. Cavaliere M. Giammarino A. Mazzone P. Olivieri V. Amorena M. (2004). Levels of polychlorinated biphenyls and organochlorine pesticides in some edible marine organisms from the Central Adriatic Sea. Chemosphere 57, 391–400 10.1016/j.chemosphere.2004.04.034 15331266

[B37] Pieper D. H. Reineke W. (2000). Engineering bacteria for bioremediation. Curr. Opin. Biotechnol. 11, 262–270 10.1016/S0958-1669(00)00094-X 10851148

[B38] Sagratini G. Buccioni M. Ciccarelli C. Conti P. Cristalli G. Giardina D. (2008). Levels of polychlorinated biphenyls in fish and shellfish from the adriatic sea. Food Addit. Contamin. 1, 69–77 10.1080/19393210802236919 24784539

[B39] Schellenberger J. Que R. Fleming R. M. Thiele I. Orth J. D. Feist A. M. (2011). Quantitative prediction of cellular metabolism with constraint-based models: the cobra toolbox v2. 0. Nat. Protoc. 6, 1290–1307 10.1038/nprot.2011.308 21886097 PMC3319681

[B40] Storelli M. Barone G. Marcotrigiano G. (2007). Polychlorinated biphenyls and other chlorinated organic contaminants in the tissues of mediterranean loggerhead turtle caretta caretta. Sci. Total Environ. 373, 456–463 10.1016/j.scitotenv.2006.11.040 17239426

[B41] Taffi M. Paoletti N. Liò P. Tesei L. Pucciarelli S. Marini M. (2014). Estimation and Modelling of PCBs Bioaccumulation in the Adriatic Sea Ecosystem. arXiv:1405.6384.

[B42] van Oevelen D. Van den Meersche K. Meysman F. Soetaert K. Middelburg J. Vézina A. (2010). Quantifying food web flows using linear inverse models. Ecosystems 13, 32–45 10.1007/s10021-009-9297-6

[B43] Williams T. D. Turan N. Diab A. M. Wu H. Mackenzie C. Bartie K. L. (2011). Towards a system level understanding of non-model organisms sampled from the environment: a network biology approach. PLoS Comput. Biol. 7:e1002126 10.1371/journal.pcbi.1002126 21901081 PMC3161900

